# Retraction Note: The Role of Scleraxis in Fate Determination of Mesenchymal Stem Cells for Tenocyte Differentiation

**DOI:** 10.1038/s41598-018-34658-3

**Published:** 2018-11-06

**Authors:** Yonghui Li, Melissa Ramcharan, Zuping Zhou, Daniel J. Leong, Takintope Akinbiyi, Robert J. Majeska, Hui B. Sun

**Affiliations:** 1 Leni and Peter W. May Department of Orthopedics, Mount Sinai School of Medicine, New York, NY 10029 USA; 2 Department of Biomedical Engineering, City College of New York, New York, NY 10031 USA; 3 Department of Orthopedic Surgery, Albert Einstein College of Medicine, Bronx, NY 10461 USA; 4 Department of Radiation Oncology, Albert Einstein College of Medicine, Bronx, NY 10461 USA; 5 Present Address: Center for Stem cell and Regenerative Medicine Guangxi Normal University, Guilin, 541004 China

Retraction of: *Scientific Reports* 10.1038/srep13149, published online 20 August 2015

The Authors are retracting this Article.

During an Inquiry into an allegation of research misconduct at the Icahn School of Medicine at Mount Sinai, where the research was conducted, it was determined that the “BMP-12 + Stretch” panel depicted in Figure 6B had been falsified. As a result, the graphs based upon Fig. 6B were also rendered inaccurate. Since these data are critical to certain main conclusions of the study, and the authors feel they cannot guarantee the accuracy of those conclusions, a retraction of the paper is warranted.

Melissa Ramcharan, Daniel J. Leong, Takintope Akinbiyi, Robert J. Majeska and Hui B. Sun agree to the retraction. Yonghui Li and Zuping Zhou could not be reached.

